# Quantitative microbial risk assessment of haemolytic uremic syndrome associated with Argentinean kosher beef consumption in Israel

**DOI:** 10.1371/journal.pone.0290182

**Published:** 2023-08-17

**Authors:** Victoria Brusa, Sergio Dolev, Marcelo Signorini, Gerardo Leotta

**Affiliations:** 1 IGEVET—Instituto de Genética Veterinaria “Ing. Fernando N. Dulout” (UNLP-CONICET LA PLATA), Facultad de Ciencias Veterinarias UNLP, La Plata, Argentina; 2 Veterinary Services and Animal Health, Ministry of Agriculture and Rural Development, Tel Aviv, Israel; 3 IdICaL–Instituto de Investigación de la Cadena Láctea (INTA–CONICET). EEA Rafaela, Instituto Nacional de Tecnología Agropecuaria (INTA), Santa Fe, Argentina; 4 ICYTESAS—Instituto de Ciencia y Tecnología de Sistemas Alimentarios Sustentables (INTA-CONICET), Buenos Aires, Argentina; University of Zambia School of Veterinary Medicine, ZAMBIA

## Abstract

The aim of this study was to perform a quantitative microbial risk assessment (QMRA) of Shiga toxin-producing *Escherichia coli* hemolytic uremic syndrome (STEC-HUS) linked to the consumption of Kosher beef produced in Argentina and consumed in Israel in children under 14 years. A probabilistic risk assessment model was developed to characterize STEC prevalence and contamination levels in the beef supply chain (cattle primary production, cattle transport, processing and storage in the abattoir, for export and at retail, and home preparation and consumption). The model was implemented in Microsoft Excel 2016 with the @Risk add-on package. Results of 302 surveys with data collected in Israel were as follows: 92.3% of people consumed beef, mostly at home, and 98.2% preferred levels of cooking that ensured STEC removal from the surface of beef cuts. The preferred degree of ground beef doneness was “well-done” (48.2%). Cooking preference ranged from red to “medium-well done” (51.8%). Median HUS probability from Argentinean beef cut and ground beef consumption in children under 14 years old was *<*10^−15^ and 8.57x10^-10^, respectively. The expected average annual number of HUS cases and deaths due to beef cut and ground beef consumption was zero. Risk of infection and HUS probability correlated with salting effect on *E*. *coli* count, processing raw beef before vegetables, ways of storage and refrigeration temperature at home, joint consumption of salad and beef cuts, degree of beef doneness and cutting board washing with detergent after each use with beef and vegetables. The STEC-HUS risk in Israel from consumption of bovine beef produced in Argentina was negligible. The current QMRA results were similar to those of previous beef cut consumption QMRA in Argentina and lower than any of the QMRA performed worldwide in other STEC-HUS linked to ground beef consumption. This study confirms the importance of QMRA to estimate and manage the risk of STEC-HUS from beef consumption. The impact variables identified in the sensitivity analysis allowed us to optimize resources and time management, to focus on accurate actions and to avoid taking measures that would not have an impact on the risk of STEC-HUS.

## Introduction

Hemolytic uremic syndrome (HUS) is a group of diseases characterized by the triad of nonimmune microangiopathic hemolytic anemia, thrombocytopenia and decreased renal function [1]. Several classifications have been proposed for HUS, such as infection-induced HUS (mainly caused by Shiga toxin producing *Escherichia coli* (STEC-HUS) and less frequently by *Streptococcus pneumoniae* and a number of other microorganisms), atypical HUS and HUS with coexisting diseases [2–4].

The presentation of STEC-HUS epidemiological data is uneven because surveillance strategies differ [5]. Some countries report STEC-HUS cases every 100,000 people, as is the case of New Zealand (0.8), Argentina (0.6), China (0.57), Ireland (0.54) and France (0.49) [4, 6–8]. In addition, STEC-HUS prevalence is reported per 100,000 children under 5 years (Argentina, 5.95; Belgium, 4.5; France, 3.1; New Zealand, 2.6; the US, 1.4; China, 0.38) [4, 6–11]. The incidence of HUS is lower in Israel than in most countries, especially because STEC-HUS is very rare, accounting for 0.01 cases per 100.000 children under 18 years old [3].

A wide range of animal species and foods have been identified as STEC reservoirs [12]. The relative importance of selected sources and transmission pathways for STEC infections at regional or global level has been assessed using outbreak data [11, 13], case-control studies of sporadic infections [14, 15], microbial subtyping [16] and expert elicitations [17]. A recent study summarizes evidence on risk factors for sporadic STEC infection through the meta-analysis of outcomes from case-control studies [18]. The main risk factors identified were foreign travel, contact with ill people, farm animals or their environment, food consumption and exposure to untreated drinking water. Regarding foodborne transmission, the consumption of meat and dairy products, especially undercooked, appeared as a risk factor in all the studied populations.

The prevalence of STEC in cattle has been reported in several countries, including Argentina [19–23]. Shiga toxin (*stx*) detection and STEC isolation from hides, carcasses, beef cuts, ground beef and hamburgers have also been evaluated [24–29]. However, the presence of STEC in cattle or meat products should not be interpreted as a direct risk to meat consumers. Quantitative microbial risk assessment (QMRA) models should be performed to establish the risk of HUS from beef consumption [22, 30–33].

The transfer of STEC to beef may occur at different stages of processing. In this sense, abattoirs are the first step in the production chain where beef may be contaminated [34–36]. The current preventive systems comply with the hazard analysis and critical control point (HACCP) principles to avoid contamination with hazards such as STEC. In the case of Kosher slaughter, the method of slaughtering animals for meat according to the Jewish law, the presence of defects is verified according to religious definitions [37]. If the carcass qualifies as Kosher, beef must go through the salting process to remove any blood remainings [38].

Israel imports some 100,000 tons of Kosher beef each year. In 2021, Argentina exported 30,707 tons to Israel from abattoirs applying HACCP (IPCVA, pers comm). In 2019, although the risk of occurrence of STEC illnesses and HUS due to beef consumption was not estimated with a QMRA, the State of Israel implemented the zero tolerance criteria for the top 7 STEC serogroups (O26, O45, O103, O111, O121, O145 and O157) on beef products.

The aim of this study was to perform a QMRA of STEC-HUS associated with the consumption of Kosher beef produced in Argentina and consumed in Israel in children under 14 years.

## Materials and methods

### Study design

**Fig 1:**
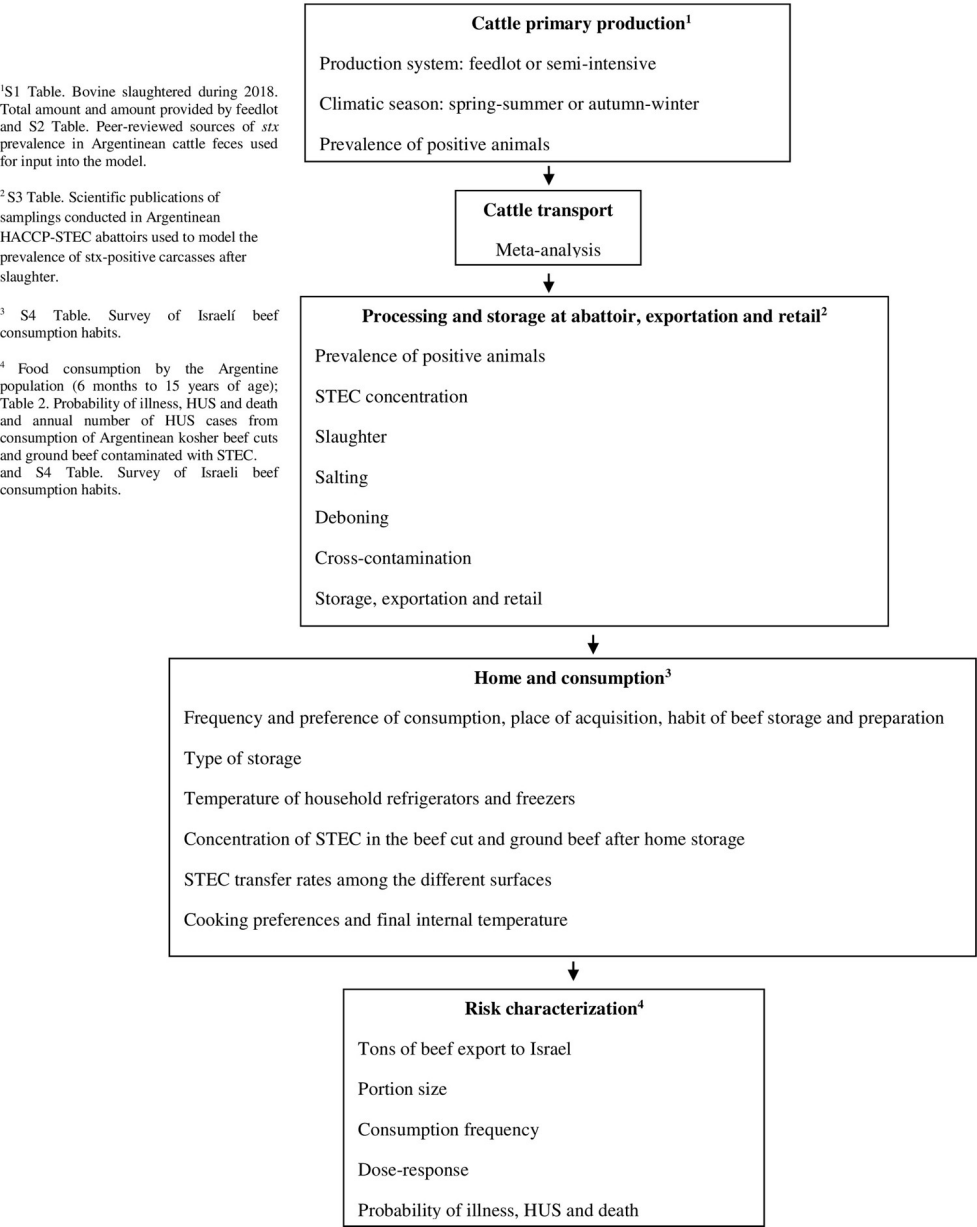
Beef supply chain conceptual model and relevant input variables. ^1^ S1, S2 Tables, ^2^ S3 Table, ^3^ S4 Table, ^4^ Table 2 and S4 Table.

The prevalence and contamination levels of STEC through the beef supply chain were characterized using a probabilistic risk assessment model (Fig 1). The beef supply chain comprised four production modules: cattle primary production, cattle transport, processing and storage in the abattoir, for export and at retail, and home preparation and consumption. The modules were used to model two beef products: ground beef (any foodstuff containing ground beef, excepting commercial hamburgers) and intact beef cuts. The model was implemented in Microsoft Excel 2016 with the @Risk add-on package (version 7.5, Palisade Corporation, New York, USA) using inputs derived from data collected in Argentina and Israel and information gathered from experts, whenever possible. A Monte Carlo simulation with Latin Hypercube Sampling was used to assess all potential scenarios. Each simulation performed 5,000 iterations of the model, which allowed to achieve an adequate level of convergence (*<*1%). Model outputs were estimated as risk per serving of contaminated beef and population risk (median and 95.0% confidence intervals). The validity of the model was analyzed by comparing the predicted number of HUS cases with data published by Alfandary et al. [3].

### Hazard identification

Shiga toxin-producing *Escherichia coli* (STEC) comprise a group of zoonotic food- and waterborne pathogens whose main virulence factor is the Shiga toxin (Stx) codified by *stx* genes [39]. The optimum temperature for STEC growth ranges between 35 °C and 42 °C; their growth is retarded at 7 °C and they continue to be viable but without developing at -20 °C. For STEC survival, water activity should be between 0.95 and 0.99 [39, 40]. Although optimum pH for STEC growth is between 6.5 and 7, they can survive in the 2.5–9.0 pH range. STEC growth is retarded above 2.5% NaCl at 37 °C and inhibited by 8.5% NaCl. The amount of salt required for STEC inhibition reduces as other factors such as temperature and pH become more appropriate for STEC growth [40]. Due to intrinsic factors such as rich nutrients, high water activity and pH, beef provides favorable conditions for STEC development [41, 42].

All STEC were included in the model, assuming a similar pathogenic potential. This definition considered the worst scenario, since all the analyses in samplings from Argentinean abattoirs exporting to Israel were negative for Top 7 STEC [43]. The STEC prevalence at different production stages of the beef supply chain in Argentina was obtained by screening results of *stx* genes and/or STEC isolation reported in the literature (S1–S3 Tables).

### Hazard characterization

The relationship between the ingested dose of STEC from beef consumption and the probability of health endpoints of interest was described using a dose-response model. The probability of illness (*Pill*) was estimated using a Beta-Poisson model relating the ingested dose of the pathogen and the probability of illness [44, 45]. The variability in parameters *α* and *β* was modelled using PERT distributions based on the 5%, 50% and 95% percentiles estimated by Teunis et al. [45].

The probability of evolution to HUS (*PHUS|ill*) of all STEC cases (0.3%) and HUS mortality rate (*Pmort|HUS*) (3/51 cases) were estimated from the data reported for the Israel region [46] and Israel [3].

### Exposure assessment

The four production modules of the beef supply chain were characterized by inputs (Fig 1). They were connected so that output distributions from each module served as inputs to the next module or as final outputs of the estimated ingested STEC dose (CFU) per serving portion (Table 1).

**Table 1:** Input parameters used in the risk assessment model of STEC due to beef consumption.

Variable	Symbol	Unit	Equation/Distribution	Reference
**1. *Cattle primary production***				
Proportion of animals slaughtered in different seasons (autumn-winter *vs*. spring-summer)	*P*_*(Se)*_	Probability	∼*Beta* [(2571435 + 1); (5430848–2571435 + 1)]	S1 Table
Proportion of animals slaughtered according to the production system (feedlot *vs*. semi-intensive system)	*P*_*(PS)*_	Probability	∼*Beta* [(805837 + 1); (5430848–805837)]	S1 Table
*stx* prevalence in animals slaughtered in autumn-winter and from feedlot production systems	*P*_*(1)*_	Probability	∼*Beta* (0 + 1; 6–0 + 1)	S2 Table
*stx* prevalence in animals slaughtered in autumn-winter and from semi-intensive production systems	*P*_*(2)*_	Probability	∼*Beta* (592 + 1; 1980–592 + 1)	S2 Table
*stx* prevalence in animals slaughtered in spring-summer and from feedlot production systems	*P*_*(3)*_	Probability	∼*Beta* (3 + 1; 18–3 + 1)	S2 Table
*stx* prevalence in animals slaughtered in spring-summer and from semi-intensive production systems	*P*_*(4)*_	Probability	∼*Beta* (401 + 1; 1865–401 + 1)	S2 Table
**2. *Cattle transport***				
Change in *stx* prevalence due to transport	*Eff*_*(Tr)*_	*Odds Ratio*	∼*PERT* (0.561; 1.028; 1.882)	[47–54]
*stx* prevalence in beef cattle after transport from farm to abattoir	*P*_*(Tr)*_	Prevalence	$\frac{\left(Prevalence\;\;\times \;Ef(Tr)\right)}{\left((1-\;Prevalence\;)\;+\;(Prevalence\;x\;Ef(Tr))\right)}$ Where “Prevalence” is *P(1)*, *P(2)*, *P(1)* or *P(4)*	
**3. *Processing and storage in the abattoir, exportation and retail***				
Change in *stx* prevalence due to slaughter	*TT*_*(s)*_	*Odds ratio*	* $\frac{∼BETA(625\;+\;1;3027\;-\;678\;+\;1)}{PTr}$ *	S3 Table
STEC prevalence in carcasses	*P*_*(c)*_	Prevalence	$\frac{\left(P(Tr)\;\times \;TT(s)\right)}{\left((1\;-\;P(Tr))\;+\;(P(Tr)\;x\;TT(s))\right)}$	
STEC concentration in carcass	*C*_*(c)*_	Log CFU/100cm^2^	∼*Normal* (2.367; 0.89 (*Truncated* (0.18; 5.06)))	[55]
Temperature in abattoirs	*Temp*_*(a)*_	°C	∼*PERT* (0; 1; 3)	Industry communication
Storage time in abattoirs	*Ti*_*(a)*_	h	∼*Triangular* (24; 27; 30)	Industry communication
STEC growth during the storage period	*C*_*(stg)*_	Log CFU/100cm	**Bacterial growth equation reported by Huang et al.** [56] **$C(alm)\;=\;C(c)\;+\;\alpha (t)\;-\;ln\left[1\;-\;\frac{1\;-\;e^{\alpha (t)}}{e^{Ym-C(f)}}\right]$** Where: $\alpha (t)\;=\;\mu \;\times \;Temp(a)\;+\;\frac{\mu }{k}\;\times \;\left[e^{-kTemp(a)}-1\right]$ $k\;=\;0.00658\;+\;\frac{1.941}{1\;+\;\text{exp}[-0.8137\;\times \;(\;\text{Temp}\;(a)\;-\;22.4)]}$ *Ym* = 8.53 × [1 –*exp* (- 0.108 × *Temp*(*a*))] $\sqrt{\mu =}∼Normal(0.0901;0.004)\;\times \;(T(a)\;-\;(∼Normal(6;1)))$	[56]
Salting effect on *E*. *coli* count	*Eff*_*(sal-ec)*_		∼*Pert* (0; 0.93; 9.6)	[43]
*E*. *coli* concentration in salting forequarters	*C*_*(fq-sal)*_		∼*C* (*stg*) × *Eff* (*sal*–*ec*)	
Salting effect on *stx*	*Eff*_*(sal-stx)*_		$∼\frac{BETA(8\;+\;1;120\;-\;8\;+\;1)\;\times \;Beta(110\;+\;1;120\;-\;110\;+\;1)}{BETA(10\;+\;1;120\;-\;10\;+\;1)\;\times \;Beta(112\;+\;1;120\;-\;112\;+\;1)}$	[43]
*stx* prevalence in salting forequarters	*P*_*(fq-sal)*_		$∼\frac{P(mr)\times Eff(sal-stx)}{\left(1-P(c))+(P(c)\times Eff(sal-stx\right)}$	
Change in STEC prevalence due to deboning process	*OR*_*(deb)*_	*Odds ratio*	* $\frac{∼BETA(16\;+\;1;\;216\;-\;16\;+\;1)}{BETA(8\;+\;1;\;120\;-\;8\;+\;1)}$ *	[43]
STEC in beef cuts at abattoir	*P*_*(bc-a)*_	Prevalence	$\frac{\left(P(bb-sal)\times OR(deb)\right)}{\left(1-P(bb-sal))+(P(bb-sal)\times OR(deb)\right)}$	
Chilled beef cuts storage temperature	*Temp*_*(ch-bc)*_	°C	∼*PERT* (0.2; 0.4; 0.5)	Industry communication
Chilled beef cuts storage time	*Ti*_*(ch-bc)*_	Hours	∼*Uniforme* (80; 85) × 24	Industry communication
Frozen beef cuts storage temperature	*Temp*_*(fr-bc)*_	°C	∼*PERT* (– 40; – 20.1; –2)	Industry communication
Frozen beef cuts storage time	*Ti*_*(fr-bc)*_	Horas	∼*Uniforme* (80; 450) × 24	Industry communication
STEC growth in beef cuts during storage	*C*_*(bc)*_	Log CFU/cm^2^	Growth equation reported by Huang et al. [56]	[56]
Surface area per gram of beef cuts	*Sa*	cm^2^/g	∼*Uniforme* (0.1; 0.5)	[57]
Grams in 100 cm2 of beef cuts	*Gcm* ^*2*^	Grams	$\frac{100}{Sa}$	
Final STEC concentration in beef cuts at retail in Israel	*FC*_*(bc-g)*_	CFU/g	$\frac{C(bc)}{Gcm2}$	
**4. Home**				
**4.a.- Beef cut**				
Storage at home	*Stg*_*(Hom)*_		∼*Beta* (78 + 1; 216–78 + 1)	S4 Table
Temperature of household refrigerators	*Temp*_*(re)*_	°C	∼*Trinagular* (-1.5; 6.1; 16.1)	[58, 59]
Temperature of household freezers	*Temp*_*(fr)*_	°C	∼*Trinagular* (- 41.1; - 20.1; - 2)	[58, 59]
STEC concentration in beef cuts at home	*C*_*(bchome)*_	CFU/g	Growth equation reported by Huang et al. [56]	[56]
Probability of eating salad with beef cuts	*Salad*_*(bc)*_		∼*Beta* (267 + 1; 275–267 + 1)	S4 Table
Probability of preparing beef cuts before salad	*P*_*(bc-Sa)*_		∼*Beta* (55 + 1; 210–55 + 1)	
Probability of washing hands	*P*_*(WH)*_		∼*Beta* (251 + 1; 286–251 + 1)	
Probability of washing cutting board	*P*_*(Wcb)*_		∼*Beta* (176 + 1; 238–176 + 1)	
Change in STEC concentration due to washing hands	*R*_*(WH)*_	%	10^∼*Normal*(-0.2; 1.42; *Truncatedo*(2))^	
Transfer rate of STEC from beef cuts to hands	*T*_*(bc-H)*_	%	10^∼*PERT*(-0.44; 0.59; 2)^	[58–66]
STEC concentration in unwashed hands	*p*_*(nonWH)*_	CFU	(*C*(*bc*) × *T*(*bc*–*H*))/100	
Number of STEC in washed hands	*p*_*(WH)*_	CFU	(*p*(*nonWH*) × *R*(*WH*))/100	
Transfer rate of STEC from hands to faucet	*T*_*(HF)*_	%	10^∼*PERT*(–2.59; –1.08; 1.09)^	[58–66]
Number of STEC in the faucet	*p*_*(F)*_	CFU	(*p*(*nonWH*) × *T*(*HF*))/100	
Transfer rate of STEC from faucet to hands	*T*_*(FH)*_	%	10^∼*PERT*(-1.7; 0.169; 2)^	[58–66]
Number of STEC in washed hands	*p*_*(WH)*_	CFU	[(*p*(*F*) × *T*(*FH*))/100] + *p*(*WH*)	
Transfer rate of STEC from hands to salad	*T*_*(HSal)*_	%	10^∼*PERT*(-2.54; 0.21; 2)^	[58–66]
Number of STEC in salad	*p*_*(Sal)*_	CFU	In washed hands: ((*p*(*WH*) × *T*(*Hsal*))/100) In unwashed hands: ((*p*(*nonWH*) × *T*(*Hsal*))/100)	
Transfer rate of STEC from beef cuts to cutting board	*T*_*(bc-cb)*_	%	10^∼*PERT*(0.48; 1.05; 1.49)^	[58–66]
Number of STEC in unwashed cutting board	*p*_*(nonWcb)*_	CFU	(*C*(*bc*) × *T*(*bc*–*cb*))/100	
Transfer rate of STEC from cutting board to salad	*T*_*(cb-Sal)*_	%	10^∼*PERT*(–0.79; –0.43; 1.73)^	[58–66]
Number of STEC in salad	*p*_*(SanonW)*_	CFU	(*p*(*nonWcb*) × *T*(*cb*–*Sal*)/100	
Final number of STEC in salad	*FC*_*(sal)*_	CFU	*C*(*Sal*) + *p*(*Sal*) + *p*(*SanonW*)	
Cooking preference	*P*_*(cooking-bc*_ *)*		∼*Discret*({1,2,3,4,5}; {0.018; 0.114; 0.257; 0.346; 0.265})	S4 Table
Cooking temperature	*Temp*_*(cook-bc)*_	°C	∼*Uniform*(75; 90)	[67]
Cooking time	*Ti*_*(cook)*_	Minutes	According to the cooking preference and the beef cut thickness: Red: ∼*Triangular* (6; 7; 15) Medium-Red: ∼*Triangular* (8; 12; 16) Medium: ∼*Triangular* (10; 12; 17) Medium-Well done: ∼*Triangular* (14; 16; 25) Well done: ∼*Triangular* (15; 20; 30)	[68]
Decimal reduction	*D*_*(bc)*_		10^(11.22 + 0.18 × *Temp*(*cook*))^	
Number of decimal reductions	*N*_*(bc)*_		$\frac{Ti(cook)}{D(bc)}$	[69]
STEC concentration in ready-to eat beef cuts	*C*_*(bc-cons)*_	CFU/g	10^(*C*(*bchome*)—*ND*(*bc*))^	
**4.b.- Ground beef**				
Storage at home	*Stg*_*(Hom)*_		∼*Beta* (48 + 1; 268–48 + 1)	S4 Table
Temperature of household refrigerators	*Temp*_*(re)*_	°C	∼*Trinagular* (– 1.5; 6.1; 16.1)	[58, 59]
Temperature of household freezers	*Temp*_*(fr)*_	°C	∼*Trinagular* (– 41.1; – 20.1; – 2)	[58, 59]
STEC concentration in ground beef home	*C*_*(gbhome)*_	CFU/g	Growth equation reported by Huang et al. [56]	[56]
Probability of eating salad with ground beef	*Salad*_*(gb)*_		∼*Beta* (267 + 1; 275–267 + 1)	S4 Table
Probability of preparing ground beef before salad	*Gb-Sal*		∼*Beta* (55 + 1; 210–55 + 1)	
Probability of washing hands	*P*_*(WH)*_		∼*Beta* (251 + 1; 286–251 + 1)	
Probability of washing cutting board	*P*_*(Wcb)*_		∼*Beta* (176 + 1; 238–176 + 1)	
Change in STEC concentration due to washing hands	*R*_*(WH)*_	%	10^∼*Normal* (– 0.2; 1.42; *Truncado*(2))^	
Transfer rate of STEC from ground beef to hands	*T*_*(gb-H)*_	%	10^∼*PERT* (– 0.44; 0.59; 2)^	[58–66]
STEC concentration in unwashed hands	*p*_*(nonWH)*_	CFU	(*C*(*bc*) × *T*(*gbh*))/100	
Number of STEC in washed hands	*p*_*(WH)*_	CFU	(*p*(*nonWH*) × *R*(*WH*))/100	
Transfer rate of STEC from hands to faucet	*T*_*(HF)*_	%	10^∼*PERT*(–2.59; –1.08; 1.09)^	[58–66]
Number of STEC in the faucet	*p*_*(F)*_	CFU	(*p*(*nonWH*) × *T*(*HF*))/100	
Transfer rate of STEC from faucet to hands	*T*_*(FH)*_	%	10^∼*PERT*(–1.7; 0.169; 2)^	[58–66]
Number of STEC in washed hands	*p*_*(WH)*_	CFU	[(*p*(*F*) × *T*(*FH*))/100] + *p*(*WH*)	
Transfer rate of STEC from hands to salad	*T*_*(HSal)*_	%	10^∼*PERT*(–2.54; 0.21; 2)^	[58–66]
Number of STEC in salad	*p*_*(Sal)*_	CFU	In washed hands: ((*p*(*WH*) × *T*(*HSal*))/100) In unwashed hands: ((*p*(*nonWH*) × *T*(*HSal*))/100)	
Transfer rate of STEC from ground beef to cutting board	*T*_*(gb-cb)*_	%	10^∼*PERT*(0.48; 1.05; 1.49)^	[58–66]
Number of STEC in unwashed cutting board	*p*_*(nonWcb)*_	CFU	(*C*(*gb*) × *T*(*gb*–*cb*))/100	
Transfer rate of STEC from cutting board to salad	*T*_*(cb-Sal)*_	%	10^∼*PERT* (– 0.79; – 0.43; 1.73)^	[58–66]
Number of STEC in salad	*p*_*(SanonW)*_	CFU	(*p*(*nonWcb*) × *T*(*cb*–*Sal*)/100	
Final number of STEC in salad	*FC*_*(sal)*_	CFU	*C*(*Sal*) + *p*(*Sal*) + *p*(*SanonW*)	
Cooking preference	*P*_*(cooking-gb)*_		∼*Discret*({1,2,3,4,5}; {0.003; 0.011; 0.109; 0.086; 0.791})	S4 Table
Cooking temperature	*Temp*_*(cook-gb)*_	°C	Red: 54.4°C Medium-Red: 58.6°C Medium: 62.7°C Medium-Well done: 65.6°C Well done: 68.3°C	[70]
Decimal reduction	*D*_*(gb)*_		10.165 + (0.211 × *Temp*(*cook*–*gb*))	[71]
STEC concentration in ready-to eat ground beef	*C*_*(gb-cons)*_		10^(*C*(*cmg*)–*D*(*gb*))^	
**Consumption**				
**5.a.- Beef cut**				
Portion size beef cut	*PS*_*(bc)*_	Grams	∼*LogNormal*(120.8; 68.7)	[72]
Ingested dose of STEC from beef cut consumption	*Dose*_*(bc)*_	CFU	With salad: (*C*(*clcons*) × *PS*(*bc*)) + *C*(*Sal*) Without salad: (*C*(*bccons*) × *PS*(*bc*))	
**5.b.- Ground beef**				
Portion size ground beef	*PS*_*(gb)*_	Grams	∼*LogNormal*(91.9; 69.3)	[72]
Ingested dose of STEC from ground beef consumption	*Dose*_*(gb)*_	CFU	With salad: (*C*(*gbcons*) × *PS*(*gb*)) + *C*(*Sal*) Without salad: (*C*(*gbcons*) × *PS*(*cm*))	
**6.- *Dose-response module***				
Probability of illness	*P*_*(ill)*_		$1-\{1+{\left(Dose(bc)/\beta \right)}^{-\alpha }\}$ Where: *α*∼*PERT* (0.000262; 0.373; 398.9) *β∼PERT*(0.056; 39.71; 39600)	[45]
Probability of HUS	*P*_*(HUS)*_		0.003	[46]
Probability of death	*P*_*(dth)*_		∼*Beta* (3 + 1; 51–3 + 1)	[3]
Probability of HUS/illness	*P*_*(HUS|ill)*_		*P* (*ill*) × *P* (*HUS*)	
Probability of death/HUS	*P*_*(dth|HUS)*_		*P* (*HUS*|*ill*) × *P* (*dth*)	
**6.a.- Beef cut**				
Number of Argentinean kosher beef cut portions consume by Israeli population	*N*_*(portbc)*_	Number	$=\frac{\left(kg\;of\;argentine\;kosher\;beef\;exported\;to\;Israel\;\right)}{\left(\;portion\;size\;in\;kg\right)}$	
Proportion of children < 14 years of age in the total population in Israel	*Prop*_*(<14y)*_		*0*.*28*	[73]
Number of beef cut portions consumed by israelí children < 14 years old			= *N* (*portbc*) × *Prop* (< 14*y*)	
Number of cases of HUS per year due to beef cut consumption	*N*_*(HUSbc)*_	Number	*N* (*portbc*) × *P* (*HUS*|*ill*)	
**6.b.- Ground beef**				
Number of consumed portions of ground beef made from Argentine kosher beef cut by total population	*N*_*(portgb)*_	Number	$=\frac{\left(kg\;of\;argentine\;kosher\;beef\;exported\;to\;Israel\;\right)}{\left(portion\;size\;in\;kg\right)}$	
Proportion of children < 14 years of age in the total population in Israel	*Prop*_*(<14y)*_		*0*.*28*	[73]
Number of ground beef portions consumed by israelí children < 14 years old			= *N* (*portgb*) × *Prop* (< 14*y*)	
Number of cases of HUS per year due to ground beef consumption	*N*_*(HUSgb)*_	Number	*N* (*portgb*) × *P* (*HUS*|*ill*)	

#### Cattle primary production

The model reported by Brusa et al. [22] was used, ‘except for the “age of animals”, which was not considered as category because only adult animals (*>*18 months) slaughtered for export to Israel were included. This classification resulted in four different production scenarios (Table 1). The proportion of animals in each season (*P*_*(Se)*_) was modelled using 2018 cattle census data [74] (S2 Table). The probability that a slaughtered animal belonged to a feedlot or semi-intensive production system (*P*_*(PS)*_) was modelled using slaughter data from feedlot animals (S2 Table). The probability of occurrence of both variables (*P*_*(Se)*_ and *P*_*(PS)*_) was modelled using Beta distributions. Data describing *stx* prevalence in cattle feces were available from several peer-reviewed studies performed in Argentina (S3 Table). The combination of *P*_*(Se)*_ and *P*_*(PS)*_ allowed to model *stx* prevalence considering potential risk factors. A syllogism was used to combine the probability of occurrence of the four level combinations (*P1*, *P2*, *P3* and *P4*). Applying the method of moments [75], these data were used to determine parameters *α* and *β* of Beta distributions and to estimate *stx* prevalence in each combination of factors.

#### Cattle transport

The results of the meta-analysis reported by Brusa et al. [22] were used to incorporate the effect of cattle transport on *stx* prevalence.

#### Processing and storage at abattoir, exportation and retail

The prevalence of *stx* and STEC levels was modelled at various stages along the production process, from arrival of live cattle to beef storage at retail (Fig 1 and Table 1). All licensed Argentinean kosher abattoirs that export beef to Israel must apply HACCP for STEC (HACCP-STEC), which include the production of vacuum-packaged beef cuts. The prevalence of *stx* in carcasses was modelled using scientific publications conducted in Argentina in HACCP-STEC abattoirs (S3 Table). The odds ratio (OR) from cross-contamination during slaughtering was calculated using *stx* prevalence in carcasses and live cattle jointly for abattoirs (*TT*_*(s)*_) (S3 Table), using the following equation (Eq 1):

$$P\;=\;\frac{Pi\;x\;OR}{1\;-\;Pi\;+\;Pi\;x\;OR}$$ (1)

where *P* is the new *stx* prevalence after a specific scenario (e.g., *stx* prevalence in carcasses at abattoir) and *Pi* is the *stx* prevalence before the specific scenario (e.g., *stx* prevalence in beef cattle in the abattoir after transport) and OR is the value between the scenarios compared.

Enumeration levels of STEC were estimated by using generic *E*. *coli* counts in carcasses from abattoirs (*C*_*(c)*_) [55]. This was considered as the most conservative scenario as STEC enumeration levels are expected to be much lower than generic *E coli* counts. The levels of STEC during cold chamber storage (*C*_*(stg)*_) were estimated using the growth equation reported by Huang et al. [56].

Cold chamber temperature (*Temp*_*(a)*_*)* and storage times (*Ti*_*(a)*_) of abattoirs were provided by the participating plants (Industry communication). The growth of STEC in beef cuts, commercial hamburgers and ground beef in the cold chamber and at retail was estimated using the same equation.

#### Beef cuts

Operators, equipment, the environment and beef are sources of STEC contamination during cutting and deboning. To model the effect of salting on bovine forequarter, results of Top 7 STEC detection and isolation and of *E*. *coli* counts in abattoirs exporting kosher beef to Israel were used [43].

To estimate the prevalence of STEC in salted bovine forequarter, the effect of salting on the concentration of *E*. *coli* (*Eff*_*(sal-ec)*_) was modeled with results informed by Brusa et al. [43], using a Pert distribution. The concentration of *E*. *coli* in salted bovine forequarter (*C*_*(fq-sal)*_) was also estimated.

Salting effect on bovine forequarter was modeled (*Eff*_*(sal-stx)*_). The *stx* prevalence in salted bovine forequarter (*P*_*(fq-sal)*_) was calculated from the *stx* prevalence in carcasses stored in cold chambers (*C*_*(stg)*_) and the salting effect (*Eff*_*(sal-stx)*_) (Table 1). The OR value due to cross-contamination during deboning to obtain beef cuts (*OR*_*(deb)*_) was modelled with data obtained in Argentina by Brusa et al. [43] in exporting Argentinean kosher beef abattoirs.

The *OR*_*(deb)*_ was calculated using STEC prevalence in salted beef cuts (*P*_*(bc-a)*_) and salted bovine forequarter (*P*_*(fq-sal)*_) jointly (S3 Table), using the previously mentioned Eq 1. The STEC concentration in beef cuts (*C*_*(bc)*_*)* was modeled using the previously mentioned growth equation [56]. The Final STEC concentration in beef cuts at retail in Israel (*FC*_*(bc-g)*_) was estimated per 100 cm^2^ of beef cuts and considered as superficial contamination. To convert load per cm^2^ (log CFU/cm^2^) to load per gram of product (log CFU/g), the relationship between the two measures was estimated. According to previous estimates, a gram of beef corresponds to 0.1–0.5 cm^2^ cut surface *(Sa)* [33]. Temperature (*Temp*_*(a)*_) and storage time (*Ti*_*(a)*_) values in beef cuts were provided by the abattoirs (Table 1) (Industry communication). Considering that Argentinean kosher beef for export to Israel is shipped chilled (34%) and frozen (66%), both forms of storage were modeled until they reach consumers (National Service of Agrifood Health and Quality of Argentina pers. comm). Chilled cuts are kept for 80 to 85 days at an average temperature of 0.4 °C (variation range, 0.2 °C to 0.5 °C). Frozen cuts are kept until they reach the consumer (80 to 450 days) at an average temperature of -20.1 °C (range, -2 °C to -40°C).

#### Home and consumption

Beef consumption habits in Israel were surveyed (S4 Table) using a descriptive epidemiological design. The survey was anonymous and self-administered. It consisted of 16 closed questions with different options to evaluate frequency and preference of beef consumption, place of acquisition, habit of beef storage and preparation. Informed consent was attached regarding anonymity, non-mandatory participation and use of research results.

#### Beef cuts

The growth of STEC and cross-contamination at home (*Stg*_*(Hom)*_) were modelled as described by Brusa et al. [43]. The probability that consumers prepared salads together with beef (*Salad*), hand washing (*P*_*(WH)*_), cutting board washing (*P*_*(Wcb)*_), the effect of cooking at home on STEC concentration and the STEC concentration after cooking (C_*(bccons)*_) (CFU/g) were modeled and estimated using the survey of Israeli consumers (S4 Table), according to Brusa et al. [43].

#### Ground beef

Argentine kosher beef exported to Israel is not subjected to any processing at the outlet level and is not marketed as minced beef (Veterinary Services and Animal Health, Ministry of Agriculture and Rural Development, pers comm). Since the mincing process occurs at home, aspects related to the way of storing ground beef at home, the temperature of refrigerators and freezers, the simultaneous consumption of salads and cross-contamination during food preparation were modeled in a similar way to that described for beef cuts. The effect of cooking during the preparation of ground beef was modelled using the survey of Israeli consumers (S4 Table) [43].

### Risk characterization

The QMRA model used the specific conditions for the production of Argentinean kosher beef for export to Israel, considering the intrinsic variability and uncertainties of each process. Risk characterization was expressed as probability of HUS and death and number of HUS cases after consuming STEC-contaminated beef products. Children under 14 years were considered the target population of this study as they represent the age group with the highest STEC-HUS incidence in Israel [3]. Final exposure to STEC was estimated as the combination of the ingested dose (CFU) in a beef serving (beef cuts and ground beef) and the dose ingested during salad consumption in case both were consumed together. Due to the lack of available information about the portion size of beef cut (*PS*_*(bc)*_) and ground beef (*PS*_*(gb)*_) consumed by children under 14 years of age in Israel, information from Argentine children aged 6 to 15 years was used to model this stage (beef cut mean portion size: 120.8 g, SD: 68.7 g; Ground beef mean portion size 91.9 g, SD: 69.3 g) [72, 86]. Similarly, since the percentage of Kosher beef consumed as beef cut or minced meat in Israel is not known, both were assessed independently, i.e., assuming that all the exported Kosher beef is consumed as beef cut and as minced meat. In 2020, 29,082 tons of Argentine kosher beef were exported to Israel (National Service of Agrifood Health and Quality of Argentina pers. comm). The number of servings of Argentine kosher beef consumed by the total population in Israel was calculated with the amount of Argentine kosher beef exported to Israel in 2020 and *PS*_*(bc)*_ and *PS*_*(gb)*_. Children under 14 years of age represent 28% of the total population in Israel [73]. To calculate the number of servings of Argentine kosher beef consumed by children under 14 years of age in Israel, the number of servings of Argentine kosher beef consumed by the total population in Israel was multiplied by 0.28. The number of annual HUS cases due to beef consumption (*N*_*(HUSbc)*_, *N*_*(HUSgb)*_, *N*_*(HUSH)*_) was estimated considering the probability of acquiring the disease (*P*_*(HUS|ill)*_) and the frequency of beef consumption.

### Sensitivity analysis

The sensitivity analysis was performed using @Risk (Palisade Inc.) to identify the processing steps with the greatest impact on the risk of acquiring STEC infection and thereby identify the risk management strategies that would generate the greatest impact on public health.

## Results

### Cattle primary production

The *stx* prevalence during primary production for all production scenarios (season and production system) was 17.57% (0.80%–38.80% 95.0% CI). Results differed when *stx* prevalence was calculated for each specific scenario, as follows: 29.83% (27.84%–31.86%) in autumn-winter, 21.49% (19.70%–31.86%) in spring-summer, 25.84% (24.50%–27.20%) in semi-intensive and 15.38% (4.50%–31.20%) in feedlot production system (Table 2).

**Table 2:** Results of the estimations for the input and output variables of the risk model.

Variable	Result
***1*. *Cattle primary production***	Mean (95% CI)
*stx* prevalence in animals slaughtered in autumn-winter	29.83% (27.84%–31.86%)
*stx* prevalence in animals slaughtered in spring-summer	21.49% (19.70%–31.86%)
*stx* prevalence in animals slaughtered from semi-intensive production systems	25.84% (24.50%–27.20%)
*stx* prevalence in animals slaughtered from feedlot production systems	15.38% (4.50%–31.20%)
*stx* prevalence for all production scenarios (season and production system)	17.57% (0.80%–38.80%).
***2*. *Cattle transport***	
*stx* prevalence in beef cattle after transport from farm to abattoir	20.38% (1.11–43.08%)
***3*. *Processing and storage in the abattoir*, *exportation and retail***	
STEC prevalence in carcasses before salting	20.53% (16.96–26.53%)
STEC concentration in carcasses before salting	0.59 (0.199–0.996) log CFU/100 cm^2^
*E*. *coli* concentration in salting forequarters	0.912 (0.06–2.84) log CFU/g cm^2^
*stx* prevalence in salting forequarters	17.89% (7.42%–33.66%)
STEC prevalence in beef cuts at abattoir	19.83% (6.0%-43.0%)
Final STEC concentration in beef cuts at retail in Israel (log CFU/100 cm^2^)	0.9022 (0.06–2.79)
Final STEC concentration in beef cuts at retail in Israel (CFU/g)	2.71x10^-3^ (1.4x10^-4^–9.86x10^-3^)
**4. Home**	
**4.a.- Beef cut**	
STEC concentration in raw beef cuts at home	-1.89 (–3.79–3.59) log CFU/g
STEC concentration in ready-to eat beef cuts	<-10 log CFU/g
**4.b.- Ground beef**	
STEC concentration in raw ground beef home	-2.51 (–3.8–1.99) log CFU/g
STEC concentration in ready-to eat ground beef	-6.52 log CFU/g (–7.88 - –1.79 log CFU /g)
**6.- *Dose-response module***	
**6.a.- Beef cut**	
Probability of illness	<10^−15^ (<10^−15^–0.008)
Probability of HUS/illness	*<*10^−15^ (<10^−15^–0.003, 90.0% CI)
Probability of death/HUS	<10^−15^ (<10^−15^–0.00, 90.0% CI)
Number of cases of HUS per year due to beef cut consumption	0
**6.b.- Ground beef**	
Probability of illness	3.22x10^-7^ (2.54x10^-9^–2.02x10^-4^)
Probability of HUS/illness	8,57x10^-10^ (7,01x10^-14^–0.003, 90% CI)
Probability of death/HUS	5.87x10^-11^ (6.43x10^-15^–0.00, 90% CI)
Number of cases of HUS per year due to ground beef consumption	0

### Processing and storage at abattoir, exportation and retail

The prevalence of *stx* and the enumeration of STEC levels on carcass surfaces before salting were 20.53% (16.96–26.53%) and 0.59 (0.199–0.996) log CFU/100 cm^2^, respectively. The prevalence of *stx* and STEC concentration in salted bovine forequarters were 17.89% (7.42%–33.66%) and 0.912 (0.06–2.84) log CFU/g cm^2^, respectively. The *stx* prevalence in beef cuts was estimated after deboning (19.83%; 6.0%-43.0%). Finally, STEC concentration in packaged beef cut at retail stores in Israel was 0.9022 (0.06–2.79) log CFU/100 cm^2^ and 2.71x10^-3^ (1.4x10^-4^–9.86x10^-3^) CFU/g (Table 2).

### Home and consumption

A total of 302 surveys from Israel were collected in February and March 2021 (S4 Table). Of the total surveyed population, 92.3% consumed beef cuts once a week (64.5%; 192/298) and ground beef less than once a week (61.6%; 183/297). Of these, 75.3% and 84.7% consumed beef cuts and ground beef at home, respectively. From those who consumed beef cuts at home, 45.5% bought it at a butcher shop and 46.5% at the supermarket. Those who consumed ground beef at home bought it at a butcher shop (37.5%) and the supermarket (62.7%). At retail, beef cuts were frozen and refrigerated according to 19.6% and 74.5% of surveyed consumers. Beef cuts and ground beef are stored frozen according to 67.3% and 79.1% of surveyed consumers, respectively. The time elapsed from the purchase of beef cuts and ground beef to consumption was less than 1 week (70% and 64% of the surveyed consumers, respectively). Most consumers (98.2%) preferred levels of cooking that ensured STEC removal from the surface of beef cuts. The preferred degrees of doneness were well-done" (26.4%), “medium-well done” (34.6%) and “medium-well” (25.7%). In the case of ground beef, the preferred degree of doneness was “well-done” (48.2%) and cooking preference ranged from red to “medium-well done” (51.8%). Both beef products were consumed sometimes or always with fresh vegetables (97%; 267/275); 59.5% reported having two separate tables to prepare beef and vegetables, whereas 26% of those who used the same table for both, sometimes or never washed the table with detergent in between handling these foods. After handling beef, 87.7% of consumers reported to wash their hands and 93% reported to wash the utensils.

The STEC concentration in raw beef cuts and ground beef was -1.89 (-3.79–3.59) and -2.51 (-3.8–1.99) log CFU/g, respectively. The STEC transfer rates from beef cuts and ground beef to salad was 10.26 (0.0–30) and 16.63 (0.0–36) CFU/g, respectively (Table 2).

### Risk characterization

Median HUS probability from consumption of Argentinean beef cuts and ground beef in children under 14 years old was *<*10^−15^ (<10^−15^–0.003, 90.0% CI) and 8,57x10^-10^ (7,01x10^-14^–0.003, 90% CI), respectively (Table 2). The expected average annual number of HUS cases and deaths due to beef cut and ground beef consumption was 0.

### Sensitivity analysis

#### Beef cuts

**Fig 2:**
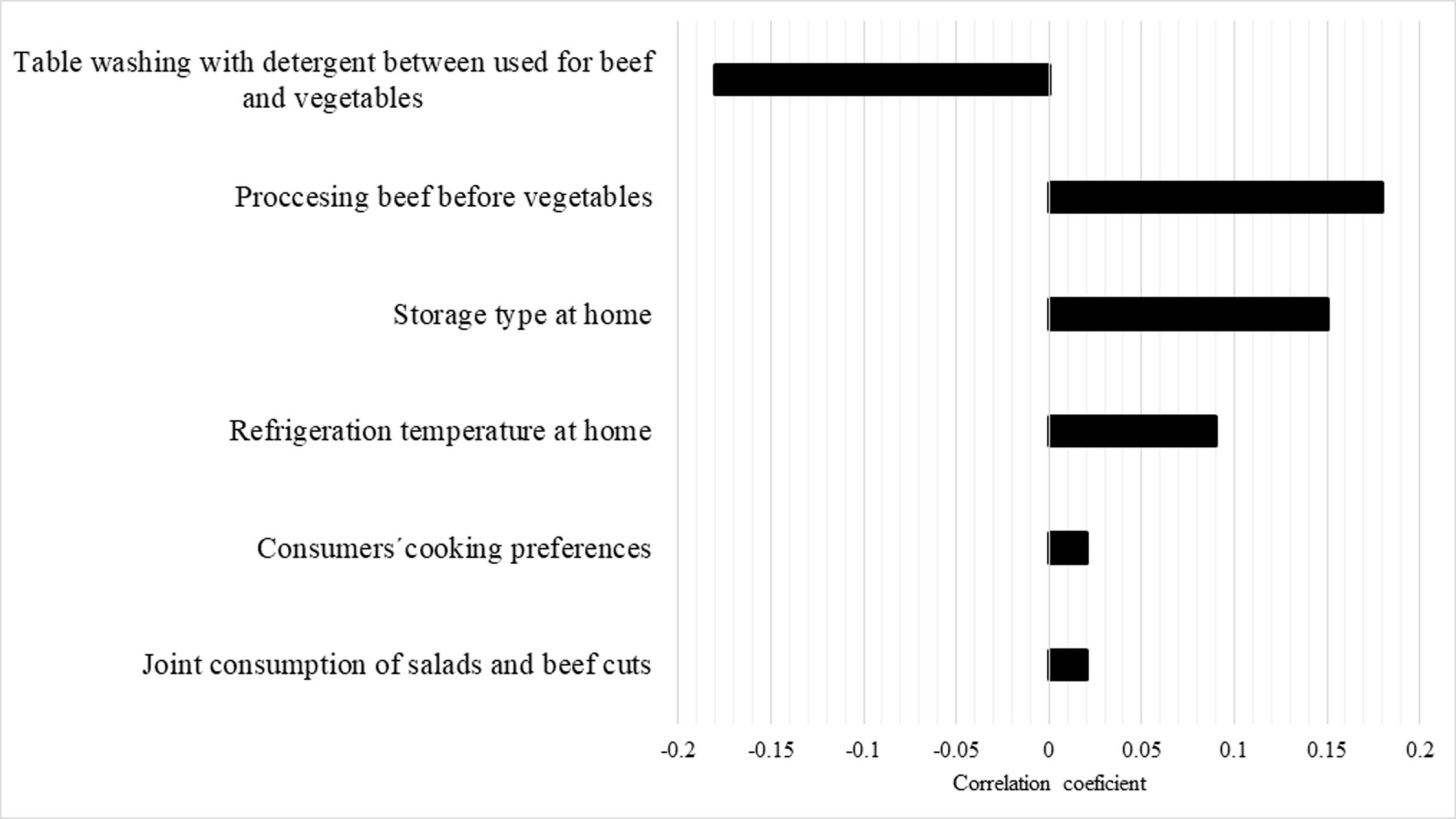
Beef cut sensitivity analysis. Model inputs on the probability of developing HUS due to beef cut consumption.

The risk of STEC infection from beef cut consumption and subsequent outcomes correlated with processing raw beef before vegetables (*r* = 0.18), storage form (*r* = 0.15), refrigeration temperature at home (*r* = 0.09), joint consumption of salad and beef cuts (*r* = 0.02), beef degree of doneness (*r* = 0.02) and table washing with detergent between use for beef and vegetables (*r* = -0.18) (Fig 2).

#### Ground beef

**Fig 3:**
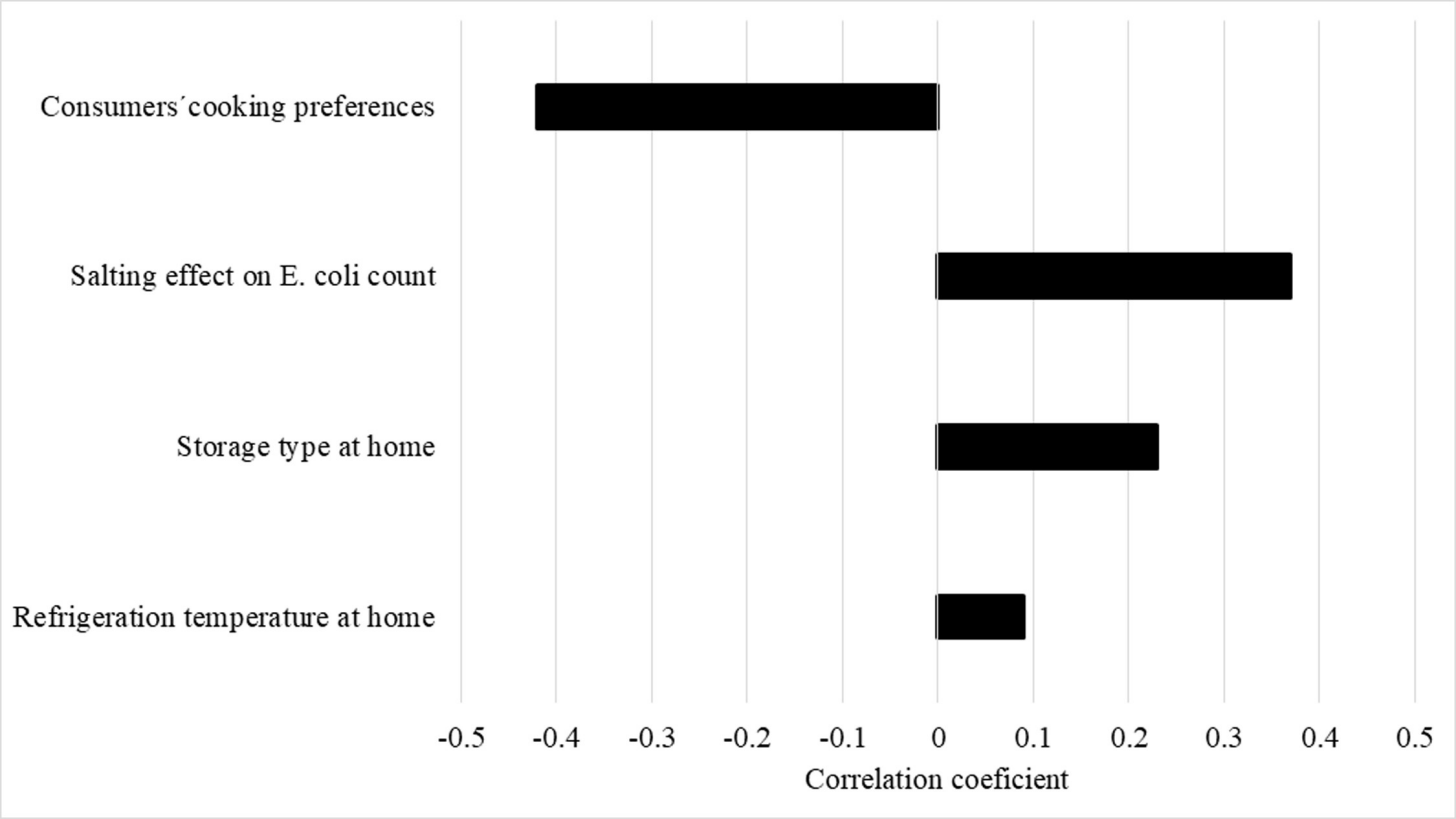
Ground beef sensitivity analysis. Model inputs on the probability of developing HUS due to ground beef consumption.

The risk of STEC infection from ground beef and subsequent outcomes correlated positively with salting effect on *E*. *coli* count (*r* = 0.37), storage form (*r* = 0.23) and temperature at home (*r* = 0.09). Ground beef cooking preference was the only input with a negative correlation (*r* = -0.42) (Fig 3).

## Discussion

The QMRA provided a mathematical representation of the beef chain from Argentina to Israel and was used to estimate current risks of STEC-HUS of Israeli children under 14 years from intact beef cut and ground beef consumption. The QMRA included all the available information throughout the Argentinean beef production chain until home consumer habits in Israel. A QMRA of HUS from intact beef cut and ground beef consumption had already been carried out in Argentina [22]. The relevance of the current QMRA is concerned with the innovative information about obtained kosher beef processing in two countries: a) one that produces the cattle and kosher beef, and b) another that imports and consumes this kosher beef. The current study includes new information, such us risk factors associated with the presence of STEC from cattle to kosher beef production; identification of risks associated with processing and storage at kosher beef abattoirs and exportation; and application of a survey to assess beef consumption habits at home in Israel.

In the present QMRA, the mean probability of HUS and death from Argentinean Kosher beef cut consumption in children under 14 years in Israel was *<*10^−15^, with an expected number of zero HUS cases per year (95.0% CI 0–0). Few studies have been published on HUS-QMRA due to intact beef cut consumption. Previous QMRAs conducted in Argentina estimated a similar probability of HUS due to beef cut consumption [22]. Other QMRAs performed in Canada estimated 2.9x10^-9^, six orders of magnitude greater than in the present study [76]. According to the bibliographic search carried out by these authors, the present study and the report by Brusa et al. [22], the only QMRA in beef cuts was conducted in Canada 9 years ago [76]. Unlike Argentinean studies, *E*. *coli* O157:H7 was the hazard evaluated in Canadian QMRAs [76].

In the present QMRA, the mean probability of HUS and death from consumption of ground beef elaborated with Argentinean Kosher beef in children under 14 years in Israel was 8.57x10^-10^ and 5.87x10^-11^, respectively, with zero HUS cases expected per year (95.0% CI 0–0). In the last 25 years, several HUS-QMRA from ground beef and hamburger consumption have been published. They were performed in Canada [76, 77], Australia [78], the Netherlands [79], the US [80, 81], and Ireland [82]. The two most recent studies were carried out in the USA and Argentina [22, 80]. The probability estimates reported in those studies (*P*_*HUS*_, 4.2×10^−9^–6.4×10^−5^; *P*_*death*_ 5.9×10^−10^–2.3×10^−6^) were between 1–6 orders of magnitude greater than in the present study. In a previous QMRA carried out in Argentina [22], the probability of HUS (5.4x10^-8^) and death (6.4x10^−9^) from ground beef consumption was two orders of magnitude greater than in Israel. Also, in contrast with the zero expected annual HUS cases in Israel, 28 HUS cases per year due to ground beef consumption were expected in Argentina. Differences in the probability and number of expected cases due to the consumption of ground beef in Israel and Argentina could be determined by the production process and consumption habits [22]. In the present QMRA, ground beef was made from Argentine vacuum-packed kosher cuts provided by abattoirs applying HACCP-STEC. However, Brusa et al. [22] considered abattoirs applying and not applying HACCP-STEC and modeled the effect of handling and cross-contamination at retail. The significantly increased bacterial load in ground beef was identified due to cross-contamination at retail [22]. The lack of sanitation standard operating procedures (SSOP) and Good Manufacturing Practices (GMP) resulted in significantly higher public health risk associated with ground beef consumption [22].

### Sensitivity analysis

Israeli consumption habits and preferences were incorporated into the model based on information from the survey carried out on the specific population. According to the present QMRA, the risk of HUS from both beef products evaluated as very low (negligible), estimating zero cases per year. However, some model input variables were identified in the sensitivity analysis, associated with higher or lower HUS risk. In coincidence with Brusa et al. [22], the joint consumption of salad and beef cuts was associated with higher HUS risk. According to surveys, the percentage of respondents who affirmed to have consumed beef cuts with vegetables was similar in Israel (64.0%) and Argentina (66.8%). Other QMRA did not consider or identify the joint consumption of salads with beef as a risk factor for HUS [30, 32].

The only variable identified with protective effect against HUS due to beef cut consumption was cutting board washing with detergent between use for beef and vegetables. In coincidence with Brusa et al. [22] (92.3%), the majority of Israeli respondents (92.8%) conducted this procedure. Cross-contamination during simultaneous beef and vegetable preparation has been previously proposed as a factor associated with illness and increased HUS risk [31, 33, 83]. Other correct food handling practices are also performed in Israel and Argentina. For example, 98.9% and 99.5% of Israeli and Argentinean people reported washing knives or other utensils used to process raw beef with detergent after use, respectively [22]. In the same way, after handling beef, 97.8% and 88.7% of Argentine and Israeli consumers reported washing their hands, respectively [22]. In contrast, 68.6% of New Zealander consumers reported washing their hands, and 41% and 28% of New Zealand respondents used knives and kitchen surfaces in a manner that could allow cross contamination [84].

In agreement with other authors, storage type and temperature at home were identified as having a positive correlation in the sensitivity analysis [22, 79]. The effect of hygiene measures, such as cutting board surface sanitization, the transference between contaminated surfaces with STEC (including hands) and vegetables and beef correct storage have also been studied [85–87].

In beef cuts, STEC contamination is superficial and can be easily destroyed by cooking since STEC are not heat-resistant [22]. Exposure to the recommended cooking temperatures eliminates STEC from beef cut surfaces [88]. However, ground beef requires thorough cooking to ensure removal of STEC from the entire product [89]. According to surveys, 1.8%, 0.7% and 0.3% of Israeli, New Zealand and Argentine consumers prefer to consume beef cuts "raw" [22, 84]. In the present QMRA, beef cut cooking preferences had a positive correlation with HUS in the sensitivity analysis. This variable was not identified by Brusa et al. [22]. Surveys conducted in New Zealand, Argentina, Ireland, Israel and Norway reported that 82.2%, 79.1%, 65.0%, 48.2% and 45.7% of consumers ate ground beef or hamburgers well-done [22, 84, 90, 91]. The same as in other risk assessments, ground beef cooking degree was identified in the sensitivity analysis and was the only input with a negative correlation with STEC-HUS probability [22, 31, 76, 77, 92].

Brusa et al. [22], reported that the impact of consumers´ habits during food preparation at home was lower than other variables in different stages of the beef chain. The present QMRA agrees with Brusa et al. [22] in that the primary production variables did not impact in the risk of HUS for any beef product. However, only one input (salting effect on *E*. *coli* counts) of processing and storage in the abattoir, at export and retail was identified in the sensitivity analysis for all the evaluated products. Few studies have evaluated the effect of salting on *E*. *coli* counts in bovine forequarter. The present QMRA applied results published by Brusa et al. [43], who did not identify statistically significant differences due to the salting effect. This could explain why this variable showed a positive correlation with the risk of HUS. More studies evaluating the salting effect could be useful to improve the present QMRA. On the other hand, the finding of several input variables in the abattoir stage in the sensitivity analysis by Brusa et al. [22] could be due to the fact that two beef abattoir categories corresponding to two different sanitary standards were modeled. However, in the present QMRA, only one type of abattoir authorized to export to Israel and with high sanitary standards was modeled.

The published information about HUS cases around the world is scarce [22]. Few primary studies and notifiable disease data from different World Health Organization (WHO) regions, and population estimates on exposure, age distribution and clinical course of illness are available [46]. Sometimes, the available epidemiological data do not discriminate HUS cases from other STEC illnesses (diarrhea, bloody diarrhea, hemorrhagic colitis), making it difficult to interpret and compare information, such as little quantitative information from countries and regions, paucity of data for incidence and DALY estimates, and lack of specification of number of outbreaks versus sporadic cases of disease [93, 94].

In Israel, the mean annual incidence of pediatric HUS is 1.5 cases per million/year. The annual incidence of STEC-HUS is 0.02 cases per 100,000 children, and the annual incidence of HUS associated with STEC was 0.01 cases per 100,000 children. Routine surveys conducted by the Israeli Ministry of Health revealed a low prevalence of STEC isolation in stool samples (<10 cases per year) [3]. According to Alfandary et al. [3], a possible explanation for the low incidence of STEC-HUS in Israel is the low level of consumption of beef compared to chicken meat, assuming that the endemicity of STEC-HUS in Argentina is due to the high consumption of beef. Beef consumption in Argentina, is 47.8 kg/person/year [95] and 16 kg/person/year (Veterinary Services and Animal Health, Ministry of Agriculture and Rural Development, pers comm) in Israel. However, the analysis would not be so simple and linear. In Argentina, the report of HUS cases is mandatory [96] and the annual incidence rate of HUS in the general population in 2021 was 0.6 cases per 100,000 inhabitants [6]. In Argentina only 4 (0.07%) STEC-HUS cases were associated with beef consumption in a period of 13 years (2002–2015) [97]. According to the last QMRA carried out in Argentina, it is estimated that only 10% of HUS cases would be due to the consumption of beef [22], with ground beef being the meat product with the highest risk. In this context, the highest prevalence of STEC-HUS in Argentina is in children ≤1 year of age, whose consumption of ground beef is low [72]. Therefore, the endemicity of STEC-HUS would not be associated directly and solely with the consumption of beef [98–100]. It is therefore necessary to consider HUS transmission in a transdisciplinary way, consolidate epidemiological studies (outbreaks and cases) and, if possible, perform QMRA to manage risks with scientific evidence.

## Conclusion

According to our study, STEC-HUS risk in Israel due to the consumption of bovine beef produced in Argentina is negligible. Estimates in the present assessment are similar to those quoted in the beef cut consumption QMRA performed in Argentina (Median<10^−15^). In addition, estimates in the present assessment from ground beef consumption are lower than any of the estimates quoted in other STEC-HUS risk assessments performed in Argentina, Canada, Australia and USA [22, 32, 76–78]. This study confirms the importance of making a QMRA to estimate and manage the risk of STEC-HUS from beef consumption. Knowledge of the impact variables identified in the sensitivity analysis allows optimizing resources and time, directing actions with greater certainty, as well as avoiding taking measures that will not have an impact on the risk of STEC-HUS.

## Supporting information

S1 Table Bovine slaughtered during 2018. Total amount and amount provided by feedlots.
(DOCX)

S2 Table Peer-reviewed sources of *stx* prevalence in Argentinean cattle feces used for input into the model. (DOCX)

S3 Table Scientific publications of samplings conducted in Argentinean HACCP-STEC abattoirs used to model the prevalence of *stx*-positive carcasses after slaughter. (DOCX)

S4 Table Survey of Israeli beef consumption habits. (DOCX)
