# Supplementary material for: Quantitative microbial risk assessment of haemolytic uremic syndrome associated with Argentinean kosher beef consumption in Israel
Source: PLoS One. 2023 Aug 17;18(8):e0290182. doi: 10.1371/journal.pone.0290182 (PMC10434954; doi:10.1371/journal.pone.0290182)
Supplement: S1 Table — Total amount and amount provided by feedlots. (DOCX) [file pone.0290182.s001.docx]

**S1 Table. Bovine slaughtered during 2018. Total bovine amount and bovine amount provided by feedlots.**

|  | **Steer** | | **Bull** | | **Cow** | | **All categories** | |
| --- | --- | --- | --- | --- | --- | --- | --- | --- |
| **Month** | **S-I** | **Feedlot** | **S-I** | **Feedlot** | **S-I** | **Feedlot** | **S-I** | **Feedlot** |
| Jan | 188,776 | 39,972 | 13,951 | 973 | 157,212 | 16,180 | 359,939 | 57,125 |
| Feb | 158,069 | 45,502 | 13,492 | 1,084 | 149,244 | 13,021 | 320,805 | 59,607 |
| Mar | 194,054 | 49,813 | 14,799 | 1,082 | 187,348 | 15,943 | 396,201 | 66,838 |
| Apr | 179,980 | 49,017 | 13,577 | 1,219 | 184,866 | 18,801 | 378,423 | 69,037 |
| May | 178,316 | 62,253 | 13,876 | 1,181 | 191,052 | 19,934 | 383,244 | 83,368 |
| Jun | 173,757 | 51,155 | 16,439 | 956 | 224,631 | 20,380 | 414,827 | 72,491 |
| Jul | 199,437 | 46,966 | 16,418 | 1,220 | 220,809 | 22,233 | 436,664 | 70,419 |
| Aug | 197,809 | 53,231 | 18,439 | 954 | 232,212 | 22,934 | 448,460 | 77,119 |
| Sept | 162,058 | 38,660 | 17,720 | 932 | 181,109 | 24,882 | 360,887 | 64,474 |
| Oct | 198,021 | 37,119 | 20,640 | 1,035 | 205,233 | 23,240 | 423,894 | 61,394 |
| Nov | 164,890 | 45,515 | 18,171 | 945 | 187,860 | 17,691 | 370,921 | 64,151 |
| Dec | 137,934 | 44,131 | 16,055 | 973 | 176,757 | 14,710 | 330,746 | 59,814 |
| Total | 2,133,101 | 563,334 | 193,577 | 12,554 | 2,298,333 | 229,949 | 4,625,011 | 805,837 |

S-I: Semi-intensive

[1]

**Reference**

1. IPCVA. [2019]. Available from: <http://www.ipcva.com.ar/>.
