# Supplementary material for: Quantitative microbial risk assessment of haemolytic uremic syndrome associated with Argentinean kosher beef consumption in Israel
Source: PLoS One. 2023 Aug 17;18(8):e0290182. doi: 10.1371/journal.pone.0290182 (PMC10434954; doi:10.1371/journal.pone.0290182)
Supplement: S2 Table — (DOCX) [file pone.0290182.s002.docx]

S2 Table. Peer-reviewed sources of *stx* prevalence in Argentinean cattle feces used for input into the model.

| **Season/Category/Production System** | **N** | **+** | **Reference** |
| --- | --- | --- | --- |
| **Spring-Summer/Adult/Feedlot** | 18 | 3 | [1] |
| **Spring-Summer/Adult/Extensive** | 34  720  720  247  144 | 0  180  94  73  54 | [2]  [3]  [4]  [1]  [5] |
| **Autumn-Winter/Adult/Feedlot** | 6 | 0 | [1] |
| **Autumn-Winter/Adult/Extensive** | 34  720  720  240  118  148 | 1  360  76  56  46  53 | [2]  [3]  [4]  [1]  [6]  [5] |

N= number of samples, each sample came from one animal; +: STEC-positive samples

**References**

1. Masana MO, D'Astek BA, Palladino PM, Galli L, Del Castillo LL, Carbonari C, et al. Genotypic characterization of non-O157 Shiga toxin-producing *Escherichia coli* in beef abattoirs of Argentina. J Food Prot. 2011;74(12):10. doi: 10.4315/0362-028X.JFP-11-189.

2. Favier GI, Estrada CL, Cortinas TI, Escudero ME. Detection and Characterization of Shiga Toxin Producing *Escherichia coli*, *Salmonella* spp., and *Yersinia* Strains from Human, Animal, and Food Samples in San Luis, Argentina. Int J Microb. 2014;2014:12. doi: 10.1155/2014/284649.

3. Fernandez D, Rodriguez EM, Arroyo GH, Padola NL, Parma AE. Seasonal variation of Shiga toxin-encoding genes (*stx*) and detection of *E. coli* O157 in dairy cattle from Argentina. J Appl Microbiol. 2009;106(4):8. doi: 10.1111/j.1365-2672.2008.04088.x.

4. Fernández D, Irino K, Sanz M, Padola NL, Parma AE. Characterization of Shiga Toxin-producing *Escherichia coli* isolated from dairy cows in Argentina. Lett Appl Microbiol. 2010;51:6. doi: 10.1111/j.1472-765X.2010.02904.x.

5. Tanaro JD, Galli L, Lound LH, Leotta GA, Piaggio MC, Carbonari CC, et al. Non-O157:H7 Shiga toxin-producing *Escherichia coli* in bovine rectums and surface water streams on a beef cattle farm in Argentina. Foodborne Pathog Dis. 2012;9(10):7. doi: 10.1089/fpd.2012.1182.

6. Sanz ME, Viñas MR, Parma AE. Prevalence of bovine verotoxin-producing *Escherichia coli* in Argentina. Eur Journal Epidemiol. 1998;14:5. doi: 10.1023/a:1007427925583.
