# Supplementary material for: Quantitative microbial risk assessment of haemolytic uremic syndrome associated with Argentinean kosher beef consumption in Israel
Source: PLoS One. 2023 Aug 17;18(8):e0290182. doi: 10.1371/journal.pone.0290182 (PMC10434954; doi:10.1371/journal.pone.0290182)
Supplement: S3 Table — (DOCX) [file pone.0290182.s003.docx]

**S3 Table. Scientific publications of samplings conducted in Argentinean HACCP-STEC abattoirs used to model the prevalence of *stx*-positive carcasses after slaughter.**

| **Type of sample** | **Abattoir section** | **Samples amount** | **Samples STEC positives** | **Reference** |
| --- | --- | --- | --- | --- |
| All carcass surface | Cold chamber | 811 | 73 | [1] |
| All carcass surface  Beef cut  Trimmings | Cold chamber  at final of deboning  deboning | 3205 grouped in 641  9570 grouped in 1914  3190 grouped in 638 | 37 pools  111 pools  45 pools | [2] |
| All carcass surface | slaughter pre-washing | 1350 | 506 | [3] |
| Standing animal leather  Standing animal leather  Perineum  All carcass surface | corral  stocks  post knockout  post intervention | 30  30  30  60 | 29  30  29  4 | [4] |
| All carcass surface  Beef cut | Cold chamber  Deboning | 165  714 | 5  58 | [5] |

**References**

1. Masana MO, D'Astek BA, Palladino PM, Galli L, Del Castillo LL, Carbonari C, et al. Genotypic characterization of non-O157 Shiga toxin-producing *Escherichia coli* in beef abattoirs of Argentina. J Food Prot. 2011;74(12):10. doi: 10.4315/0362-028X.JFP-11-189..

2. Brusa V, Restovich V, Galli L, Teitelbaum D, Signorini M, Brasesco H, et al. Isolation and characterization of non-O157 Shiga toxin-producing *Escherichia coli* from beef carcasses, cuts and trimmings of abattoirs in Argentina. PLoS One. 2017;12(8):16. doi: 10.1371/journal.pone.0183248.

3. Signorini M, Costa M, Teitelbaum D, Restovich V, Brasesco H, Garcia D, et al. Evaluation of decontamination efficacy of commonly used antimicrobial interventions for beef carcasses against Shiga toxin-producing *Escherichia coli*. Meat Sci. 2018;142:8. doi: 10.1016/j.meatsci.2018.04.009.

4. Brusa V, Restovich V, Signorini M, Pugin D, Galli L, Diaz VR, et al. Evaluation of intervention measures at different stages of the production chain in Argentinian exporting abattoirs. Food Sci Tech Int. 2019;25(6):6. doi: 10.1177/1082013219836326.

5. Brusa V, Restovich V, Galli L, Arias R, Linares L, Costa M, et al.. Reduction of Shiga toxin-producing *Escherichia coli*. Food Science and Technology International. 2022; 28(1):50-59. doi: 10.1177/1082013221991258. Epub 2021 Feb 7.
