# Supplementary material for: Quantitative microbial risk assessment of haemolytic uremic syndrome associated with Argentinean kosher beef consumption in Israel
Source: PLoS One. 2023 Aug 17;18(8):e0290182. doi: 10.1371/journal.pone.0290182 (PMC10434954; doi:10.1371/journal.pone.0290182)
Supplement: S4 Table — (DOCX) [file pone.0290182.s004.docx]

**S4 Table. Survey of Israeli beef consumption habits.**

| 1. **Age** | **Responded N (%)** | | **Answer N (%)** | | | | | | | | | | | | | | | | | | | | |
| --- | --- | --- | --- | --- | --- | --- | --- | --- | --- | --- | --- | --- | --- | --- | --- | --- | --- | --- | --- | --- | --- | --- | --- |
|  | 300 (99.3) | | 20 to 71 years old: 300 (99.3) | | | | | | | | | | | | | | | | | | | | |
| **2. Do you consume beef and beef products?** | **Responded N (%)** | | **Yes N (%)** | | | | | | | | | | **No N (%)** | | | | | | | | | | |
|  | 298 (98.7) | | 275 (92.3) | | | | | | | | | | 23 (7.7) | | | | | | | | | | |
| **3. How often do you consume beef products? (Number of times a week you consume them)** | **Responded N (%)** | **Answer N (%)** | | | | | | | | | | | | | | | | | | | | | |
|  |  | **0** | **<1** | **1** | | | **2** | | | **3** | | | **4** | | | **5** | | | **6** | | | | **7** |
| 3.1. Intact beef cut ^1^ | 298 (98.7) | 45 (15.1) | 58 (19.5) | 89 (29.9) | | | 50 (16.7) | | | 31 (10.4) | | | 13 (4.4) | | | 5 (1.7) | | | 2 (0.7) | | | | 5 (1.7) |
| 3.2. Food made with raw ground beef ^2^ | 297 (98.3) | 41 (13.8) | 50 (16.8) | 92 (31.0) | | | 61 (20.5) | | | 31 (10.4) | | | 6 (2.0) | | | 12 (4.0) | | | 2 (0.7) | | | | 2 (0.7) |
| **4. Where do you consume beef products? *** | **Responded N (%)** | | **Answers with multiple options* N (%)** | | | | | | | | | | | | | | | | | | | | |
|  |  |  | **Total** | | | | **Home** | | | | | | **Restaurant** | | | | | **Other** | | | | | |
| 4.1. Intact beef cut ^1^ | 280 (92.7) | | 280 (100.0) | | | | 211 (75.3) | | | | | | 54 (19.3) | | | | | 15 (5.3) | | | | | |
| 4.2. Food made with raw ground beef ^2^ | 281 (93.0) | | 281 (100.0) | | | | 238 (84.7) | | | | | | 24 (8.5) | | | | | 19 (6.8) | | | | | |
| **5. If you consume beef products at home. Where do you buy beef?*** | **Responded N (%)** | | **Answers with multiple options* N (%)** | | | | | | | | | | | | | | | | | | | | |
|  |  |  | **Total** | | | | **Warehouse** | | | | **Butcher Shop** | | | | | **Supermarket** | | | | | **Other** | | |
| 5.1. Intact beef cut ^1^ | 275 (91.0) | | 380 (100.0) | | | | 105 (1.6) | | | | 125 (45.4) | | | | | 128 (46.5) | | | | | 22 (8.0) | | |
| 5.2. Food made with raw ground beef ^2^ | 276 (91.4) | | 351 (100.0) | | | | 75 (1.6) | | | | 87 (31.5) | | | | | 173 (62.7) | | | | | 16 (5.8) | | |
| **6. How is the beef product stored in the place where you buy it?*** | **Responded N (%)** | | **Answers with multiple options* N (%)** | | | | | | | | | | | | | | | | | | | | |
|  |  |  | **Total** | | | | | **Frozen** | | | | **Chilled** | | | | | | **Other** | | | | | |
| 6.1. Intact beef cut ^1^ | 267 (87.9) | | 267 (100.0) | | | | | 53 (19.9) | | | | 199 (74.5) | | | | | | 15 (5.6) | | | | | |
| 6.2. Food made with raw ground beef ^2^ | 270 (88.8) | | 270 (100.0) | | | | | 67 (24.8) | | | | 184 (68) | | | | | | 19 (7.0) | | | | | |
| **7. How do you store beef products at home until consumption?*** | **Responded N (%)** | | **Answers with multiple options* N (%)** | | | | | | | | | | | | | | | | | | | | |
|  |  |  | **Total** | | | | | **Frozen** | | | | **Chilled** | | | | | | **Another** | | | | | |
| 7.1. Intact beef cut ^1^ | 272 (90.1) | | 272 (100.0) | | | | | 183 (67.3) | | | | 78 (28.7) | | | | | | 11 (4.0) | | | | | |
| 7.2. Food made with raw ground beef ^2^ | 278 (92.0) | | 278 (100.0) | | | | | 220 (79.1) | | | | 48 (17.3) | | | | | | 10 (3.6) | | | | | |
| **8. How long does it take from buying the beef product until consumption? *** | **Responded N (%)** | | **Answers with multiple options* N (%)** | | | | | | | | | | | | | | | | | | | | |
|  |  |  | **Total** | | **0 Day** | | | **1-3 Days** | | | **3-5 Days** | | | **5-7 Days** | | | | **Weeks** | | | | **Months** | |
| 8.1. Intact beef cut ^1^ | 268 (88.7) | | 268 (100.0) | | 25 (9.3) | | | 79 (29.5) | | | 27 (10.1) | | | 54 (20.1) | | | | 74 (27.7) | | | | 9 (3.3) | |
| 8.2. Food made with raw ground beef ^2^ | 269 (89.0) | | 269 (100.0) | | 26 (9.7) | | | 53 (19.8) | | | 35 (13.0) | | | 59 (22.0) | | | | 86 (32.0 | | | | 10 (3.7) | |
| **9. What degree of cooking does your family prefer for beef products?** | **Responded N (%)** | | **Answer N (%)** | | | | | | | | | | | | | | | | | | | | |
|  |  |  | **Red** | | | **Medium-red** | | | | **Medium-well** | | | | | **Medium-well done** | | | | | **Well-done** | | | |
| 9.1. Intact beef cut ^1^ | 280 (92.7) | | 5 (1.8) | | | 32 (11.4) | | | | 72 (25.7) | | | | | 97 (34.6) | | | | | 74 (26.4) | | | |
| 9.2. Food made with raw ground beef ^2^ | 278 (92.0) | | 2 (0.7) | | | 3 (1.1) | | | | 35 (12.6) | | | | | 104 (37.4) | | | | | 134 (48.2) | | | |
| **10. How often do you accompany beef products with fresh vegetables (green leaf salads. tomatoes)?** | **Responded N (%)** | | **Answer N (%)** | | | | | | | | | | | | | | | | | | | | |
|  |  |  | **Sometimes** | | | | | | **Always** | | | | | | | | **Never** | | | | | | |
| 10.1. Intact beef cut ^1^ | 275 (91.0) | | 91 (33.1) | | | | | | 176 (64.0) | | | | | | | | 8 (2.9) | | | | | | |
| 10.2. Food made with raw ground beef^2^ | 275 (91.5) | | 109 (39.6) | | | | | | 158 (57.4) | | | | | | | | 8 (2.9) | | | | | | |
| **11. Do you have two tables identified at home for the preparation of raw beef and vegetables?** | **Responded N (%)** | | **Answer N (%)** | | | | | | | | | | | | | | | | | | | | |
|  |  |  | **Yes** | | | | | | **No** | | | | | | | | **Unknown** | | | | | | |
|  | 297 (98.3) | | 169 (56.9) | | | | | | 113 (38.0) | | | | | | | | 15 (5.0) | | | | | | |
| **12. Do you use the tables identified for the preparation of raw beef and vegetables?** | **Responded N (%)** | | **Answer N (%)** | | | | | | | | | | | | | | | | | | | | |
|  |  |  | **Yes** | | | | | | **No** | | | | | | | | **Unknown** | | | | | | |
|  | 274 (90.7) | | 163 (59.5) | | | | | | 91 (33.2) | | | | | | | | 20 (7.3) | | | | | | |
| **13. Do you use the same table for raw beef and vegetables? In what order? *** | **Responded N (%)** | | **Answers with multiple options* N (%)** | | | | | | | | | | | | | | | | | | | | |
|  |  |  | **Total** | | | | | | **Beef and then vegetables** | | | | | | | | **Vegetables and then beef** | | | | | | |
|  | 210 (69.5) | | 210 (100.0) | | | | | | 55 (26.2) | | | | | | | | 155 (73.8) | | | | | | |
| **14. If you use the same table to process raw beef and vegetables. Do you wash the table with detergent between these foods?** | **Responded N (%)** | | **Answer N (%)** | | | | | | | | | | | | | | | | | | | | |
|  |  |  | **Sometimes** | | | | | | **Always** | | | | | | | | **Never** | | | | | | |
|  | 238 (78.8) | | 45 (18.9) | | | | | | 176 (73.9) | | | | | | | | 17 (7.1) | | | | | | |
| **15. After processing the raw beef. Do you wash your hands with detergent and/or soap?** | **Responded N (%)** | | **Answer N (%)** | | | | | | | | | | | | | | | | | | | | |
|  |  |  | **Sometimes** | | | | | | **Always** | | | | | | | | **Never** | | | | | | |
|  | 286 (94.7) | | 32 (1.0) | | | | | | 251 (87.7) | | | | | | | | 3 (1.0) | | | | | | |
| **16. Do you wash the knives or other utensils used to process raw beef with detergent after use?** | **Responded N (%)** | | **Answer N (%)** | | | | | | | | | | | | | | | | | | | | |
|  |  |  | **Sometimes** | | | | | | **Always** | | | | | | | | **Never** | | | | | | |
|  | 285 (94.4) | | 17 (5.9) | | | | | | 265 (93.0) | | | | | | | | 3 (1.0) | | | | | | |

* Answers with multiple options. Respondents were able to select more than one answer.

^1^ Anatomical beef cuts without mechanical treatments as tender.

^2^ Excepting commercial hamburgers and sausages.
